# Time trends of physical activity and television viewing time in Brazil: 2006-2012

**DOI:** 10.1186/s12966-014-0101-4

**Published:** 2014-08-15

**Authors:** Grégore I Mielke, Pedro C Hallal, Deborah C Malta, I-Min Lee

**Affiliations:** Postgraduate Program in Epidemiology, Federal University of Pelotas, Pelotas, RS Brazil; Ministry of Health, Brasília, DF Brazil; Division of Preventive Medicine, Brigham and Women’s Hospital, Harvard Medical School, Boston, MA USA

**Keywords:** Physical activity, Trends, Surveillance, Epidemiology, Developing countries

## Abstract

**Background:**

Despite recent advances in surveillance of physical activity, data on time trends of physical activity in low and middle-income countries are lacking. This study describes time trends in physical activity and television viewing between 2006 and 2012 among Brazilian adults.

**Methods:**

Data from 371,271 adult participants (18 + years) in the Surveillance System for Risk and Protective Factors for Chronic Illnesses using Telephone Survey (VIGITEL) were analysed. Time trends in leisure-time physical activity (≥ 5 days/wk; ≥ 30 min/day), transportation physical activity (using bicycle or walking for ≥ 30 minutes per day as a means of transportation to/from work) and proportion of participants spending more than three hours per day watching television were analysed. Annual changes according to sex, age and years of schooling were calculated.

**Results:**

There was an increase in leisure-time physical activity from 12.8% in 2006 to 14.9% in 2012 (annual increase of 1.9%; p < 0.001). This increase was more marked in younger participants and those with high-school education. Transportation physical activity decreased 12.9% per year (p < 0.001) from 2006 to 2008 and 5.8% per year from 2009 to 2012 (p < 0.001). The annual decline in television viewing time was 5% (p < 0.001) between 2006 and 2009 and 2% (p = 0.16) between 2010 and 2012.

**Conclusion:**

National survey data from Brazil indicate that leisure-time physical activity appears to be increasing, while television viewing time appears to be decreasing in recent years. However, transportation physical activity has been declining. These data are important for informing national public health policies.

## Background

Physical inactivity is responsible for 5.3 million deaths per year worldwide [1]. Surveillance of physical activity has progressed substantially, particularly over the past 10 years, so that survey data using comparable methodology are now available for over 120 countries. The prevalence of physical inactivity among adults, living in countries with approximately 90% of the world’s population, was estimated at 31% in a recent publication [2]. Two key limitations in the surveillance of physical activity to date are that (a) lack of data is more frequently observed in low and middle as compared to high-income countries; (b) few countries have collected data at more than one time, precluding the examination of time trends [2].

Since physical activity is essential for good health, regular monitoring of population physical activity levels is important for decision making in public health. Some high-income countries, such as the United States [3,4] and Canada [5,6], have continuous surveillance systems operating so that it is possible to monitor trends in physical activity at the population level. In Brazil, a surveillance system was implemented in 2006 to annually collect data on several risk factors and health outcomes [7]. Data on physical activity in multiple domains was obtained in this survey, the Surveillance System of Protective and Risk Factors for Chronic Diseases Telephone Survey (VIGITEL).

VIGITEL represents the first national survey to collect physical activity data. Previously, time trends of physical activity in Brazil had been evaluated at a local level only. In Pelotas, a Southern Brazilian city, the prevalence of physical inactivity, defined as not obtaining sufficient physical activity to meet current guidelines, increased sharply between 2002 and 2007, and modestly between 2007 and 2012 [8]. When examining only leisure-time physical activity, no changes were detected between 2004 and 2010, implying that declines in physical activity during this period likely occurred due to decreases in activities related to occupation, housework and/or commuting [9]. In contrast, in the state of Sao Paulo, the prevalence of physical inactivity was shown to decrease considerably between 2002 and 2008 [10].

Therefore, to provide more comprehensive information on a national level, the aim of this study was to describe time trends in physical activity and television viewing time between 2006 and 2012 in a national sample of Brazilian, examining overall patterns, physical activity in different domains, and patterns according to sex, age and education.

## Methods

### Sample selection

We analysed information from VIGITEL collected annually since 2006. VIGITEL interviewed adults (18+ years) living in 26 Brazilian state capitals and the Federal District. Details about the methods have been published elsewhere [7,11]. In summary, each year, VIGITEL obtains lists of telephone lines registered in the state capitals and Federal District by landline phone companies. Every year, a random sample of 5,000 telephone lines is sampled in each state capital. Commercial and non-operational lines are then excluded. Households holding the remaining lines in that block of 5,000 are then listed and 2,000 are sampled randomly; within each household sampled, one resident is randomly selected to be interviewed – all residents of each household aged 18+ years are listed and a table of random numbers is used to select only one per household. The sample in each year comprises approximately 2,000 adults in each city, with around 54,000 participants in the entire country per year. Due to logistic and funding aspects, the 2012 survey included approximately 1,600 and not 2,000 individuals per state capital. Table 1 describes the number of interviews and response rates in each year.

**Table 1:** **Characteristics of samples in the national Brazilian Surveillance System of Protective and Risk Factors for Chronic Diseases Telephone Survey (VIGITEL), 2006-2012**

**Indicators**	**2006**	**2007**	**2008**	**2009**	**2010**	**2011**	**2012**
Interviewed	54,369	54,251	54,353	54,367	54,339	54,144	45,448
Females (%)	53.9	53.9	53.9	53.9	53.9	53.9	53.9
Mean age (SD)	40.1 (16.2)	40.3 (16.2)	40.5 (16.2)	40.8 (16.1)	40.9 (16.3)	41.1 (16.3)	41.3 (16.4)
0-4 years of schooling (%)	21.6	20.4	19.4	18.0	16.8	17.2	16.1
Response rate (%)	90.9	92.3	94.2	97.0	97.7	97.8	94.1

### Physical activity indicators

From the start, the VIGITEL system has included questions on physical activity in four domains: leisure time, transportation, occupation and household. It also includes information about television viewing time as an indicator of sedentary behaviour. However, certain physical activity questions were modified over time. Thus, to maintain comparability, this paper is limited to analyses of only physical activity questions that remained unchanged, or only underwent minor modifications. If the latter, analyses are presented separately before and after the minor modifications for valid comparisons.

In order to construct an indicator of leisure-time physical activity, the following questions were used: 1) Have you practiced sports or exercise in the last three months?; 2) How many days do you usually practice sports/exercise per week?; 3) On these days, how much time do you spend in sports/exercise? Participants who reported practicing sports and/or exercise on at least five days per week for at least 30 minutes each day [12] were considered to be active during leisure time.

With regards to commuting physical activity, minor changes in the questions occurred after 2008. Up to 2008 the following questions were asked: 1) Do you walk or use your bicycle as a means of transportation? 2) How much time do you spend walking/biking to/from work? After 2009, the following questions were used: 1) Do you make any part of your journey to/from work walking or cycling? 2) How much time do you spend to/from work per day walking or cycling? For the present analyses, we defined transportation physical activity as using bicycles or walking as a means of transportation to/from work of at least 30 minutes per day.

Data on occupational and household physical activity were collected using multiple choice questions on walking and carrying heavy weight at work, and whether or not the respondent is responsible for the heavy cleaning of his/her household. Because these questions underwent major changes over the years, we chose not to include these domains of physical activity in our analysis of time trends.

For sedentary behaviour, the number of days and time spent watching television was collected from 2006 to 2009. From 2010 onwards, only television time in a typical day was measured. We calculated the proportion of participants spending more than three hours per day watching television as an indicator of sedentary behaviour.

### Statistical analysis

In the analyses, we used post stratification weights to account for sampling, so that findings can be extrapolated to the population of each state capital, as well as to the national population. These weights matched the socio-demographic profile of the adult population with a landline telephone in each city to the socio-demographic profile of the total adult population in the same city and year of survey, in terms of sex, age and schooling level. This matching was made possible through the use of Census 2010 data. The Rake method was used to estimate the population in each year based on census data. The use of post-stratification weights applied to the sample VIGITEL, equal to that estimated for the socio-demographic distribution of the adult population distribution [13,14].

Time trends for each physical activity indicator were stratified by sex (male; female), age (18-24; 25-34; 35-44; 45-54; 55-64; 65+ years) and years of schooling (0-4; 5-8; 9-11; 12 or more). The statistical significance of changes between years was calculated by Poisson regression, with the physical activity indicator as the outcome of interest and year of survey as the exposure. Analyses were conducted using the statistical software package Stata version 12.0 (Stata Cort., College Station, USA).

### Ethical considerations

This study was approved by the National Human Research Ethics Committee of the Brazilian Ministry of Health. Consent from each participant was obtained prior to the interview.

## Results

For the entire period included in our analyses (2006 to 2012), data from 371,271 participants were available. The lowest response rate was observed in 2006 (90.9%). From 2006 to 2012, there was a slight increase in the mean age of the population and a decrease in the proportion of participants with 0-4 years of schooling (Table 1).

**Figure 1:**
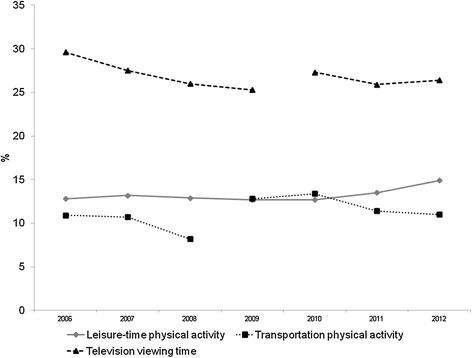
**Time trends in leisure-time and transportation physical activity and television viewing time. Brazil 2006-2012.** a-The lines for transportation physical activity and television viewing time are not continuous, with the breaks indicating the year in which minor changes occurred in the wording of questions related to each variables; b-Leisure time physical activity: practicing sports and/or exercise on at least five days per week for at least 30 minutes each day; c-Transportation physical activity: walk or bicycle from/ to work at least 30 minutes per day; d-watching television more than three hours per day.

Figure 1 presents time trends of leisure-time and transportation physical activity, as well as the proportion of adults who spent more than three hours per day watching television. There was an increase in leisure-time physical activity over time (12.8% in 2006 to 14.9% in 2012). This change represents an increase of 1.9% per year (p < 0.001). As the questions on physical activity during transportation changed slightly over time, we separately analysed the periods 2006 to 2008 and 2009 to 2012. In both periods, transportation physical activity decreased over time. From 2006 to 2008, the annual average decrease was 12.9% (p < 0.001), whereas the equivalent figure for 2009-2012 was 5.8% per year (p < 0.001). In terms of television time, there were also reductions in both periods analysed. The annual decline rate was 5% (p < 0.001) between 2006 and 2009 and 2% (p = 0.16) between 2010 and 2012.

**Figure 2:**
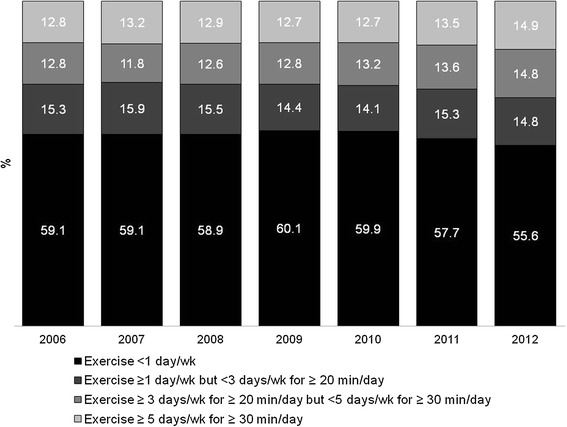
**Time trends in patterns of leisure-time physical activity.**

Figure 2 shows detailed time trends of leisure-time physical activity. For these analyses, we defined four mutually exclusive categories: (1) exercise <1 day/wk; (2) exercise ≥ 1 day/wk but <3 days/wk for ≥ 20 min/day; (3) exercise ≥ 3 days/wk for ≥ 20 min/day but <5 days/wk for ≥ 30 min/day; or (4) exercise ≥ 5 days/wk for ≥ 30 min/day. The proportion of participants reporting exercise less than once a week declined from 59.1% in 2006 to 55.6% in 2012. Combining those in the last two categories; i.e., those who exercised for at least 3 days a week for at least 20 minutes per day, the proportion of such active individuals rose from 25.6% in 2006 to 29.7% in 2012. This change represents an annual increase of 2.4% in leisure-time physical activity (p < 0.001). The proportion of individuals practicing some leisure-time physical activity, but not achieving ≥ 3 days/wk for ≥ 20 min/day remained stable over time.

Table 2 displays time trends in leisure-time physical activity according to sex, age and years of schooling. Changes were similar between men and women, but there were differences according to age. There was a large increase in leisure-time physical activity among younger adults (annual change of 4.1%; p <0.001) and small decreases in leisure-time physical activity in the two oldest groups (55+ years). The higher the schooling achieved, the higher was the increase in leisure-time physical activity over time.

**Table 2:** Time trends in leisure time physical activity* by sex, age and years of schooling- Brazil 2006-2012

**Variables**	**2006**	**2007**	**2008**	**2009**	**2010**	**2011**	**2012**	**Annual change (%)**	**p value**
Sex									
Male	14.5	15.0	14.4	14.7	14.5	15.5	16.9	+2.0	0.002
Female	11.3	11.6	11.7	11.1	11.2	11.8	13.2	+1.7	0.002
Age (years)									
18-24	15.9	17.1	16.2	16.7	16.5	18.7	21.2	+4.1	< 0.001
25-34	13.0	12.6	12.2	13.2	13.1	13.5	16.5	+3.7	< 0.001
35-44	10.1	11.5	10.9	10.2	10.5	11.2	12.6	+2.1	0.029
45-54	11.7	11.0	11.4	11.2	11.8	11.9	11.9	+0.8	0.406
55-64	14.6	14.2	14.7	12.8	12.4	13.0	12.5	-2.9	0.010
65+	12.1	13.3	13.5	12.4	11.7	12.8	12.7	-0.3	0.783
Years of schooling									
0-4	9.4	9.3	8.9	8.2	8.7	8.6	10.2	+0.2	0.856
5-8	11.1	10.4	10.8	9.7	10.2	10.7	10.3	-0.9	0.435
9-11	14.7	15.1	14.8	14.7	13.8	15.4	17.0	+1.7	0.006
12+	15.5	17.3	16.0	16.5	16.6	16.6	18.4	+1.8	0.013

*Exercise 5 days/week for at least 30 minutes/day.

Table 3 presents regression analyses results for the correlates of leisure-time physical activity in each year. Women were consistently around 20% less active than men even after adjustment for age and schooling. Interestingly, the lower levels of physical activity among older adults (65+ years) observed in the unadjusted analyses are not confirmed when adjustment for sex and schooling is performed. In fact, the least active group in leisure-time in all adjusted analyses is that of individuals aged 35-44 years. The positive association observed between leisure-time physical activity and schooling in the unadjusted analyses is confirmed in the regression models.

**Table 3:** Adjusted analysis on time trends in leisure time physical activity* by sex, age and years of schooling- Brazil 2006-2012

**Variables**	**2006**	**2007**	**2008**	**2009**	**2010**	**2011**	**2012**
Sex							
Male	1.00	1.00	1.00	1.00	1.00	1.00	1.00
Female	0.78 (0.72-0.85)	0.77 (0.71-0.85)	0.81 (0.74-0.88)	0.76 (0.69-0.82)	0.77 (0.70-0.84)	0.76 (0.70-0.83)	0.79 (0.72-0.86)
Age (years)							
18-24	1.00	1.00	1.00	1.00	1.00	1.00	1.00
25-34	0.84 (0.74-0.96)	0.76 (0.67-0.86)	0.77 (0.68-0.88)	0.83 (0.73-0.94)	0.82 (0.71-0.94)	0.74 (0.65-0.83)	0.80 (0.70-0.91)
35-44	0.70 (0.61-0.79)	0.76 (0.67-0.86)	0.75 (0.67-0.85)	0.70 (0.61-0.80)	0.72 (0.62-0.84)	0.63 (0.58-0.76)	0.65 (0.56-0.74)
45-54	0.84 (0.73-0.97)	0.77 (0.67-0.86)	0.83 (0.73-0.95)	0.81 (0.70-0.93)	0.83 (0.71-0.97)	0.73 (0.64-0.83)	0.65 (0.56-0.75)
55-64	1.11 (0.95-1.31)	1.07 (0.93-1.24)	1.14 (0.98-1.32)	0.99 (0.86-1.15)	0.93 (0.79-1.08)	0.85 (0.74-0.98)	0.71 (0.61-0.82)
65+	1.00 (0.85-1.18)	1.10 (0.94-1.28)	1.15 (0.98-1.34)	1.07 (0.92-1.25)	0.98 (0.82-1.17)	0.92 (0.79-1.07)	0.78 (0.67-0.92)
Years of schooling							
0-4	1.00	1.00	1.00	1.00	1.00	1.00	1.00
5-8	1.20 (1.01-1.42)	1.15 (0.97-1.36)	1.27 (1.07-1.50)	1.19 (0.99-1.43)	1.17 (0.96-1.42)	1.22 (1.02-1.47)	0.94 (0.78-1.13)
9-11	1.61 (1.38-1.87)	1.71 (1.48-1.97)	1.81 (1.58-2.09)	1.85 (1.58-2.17)	1.59 (1.33-1.88)	1.75 (1.48-2.07)	1.48 (1.25-1.74)
12+	1.69 (1.45-1.98)	1.94 (1.68-2.26)	1.95 (1.68-2.26)	2.08 (1.77-2.44)	1.90 (1.60-2.27)	1.90 (1.60-2.25)	1.60 (1.35-1.90

*Exercise 5 days/week for at least 30 minutes/day.

Major changes were observed for transportation physical activity – declines were observed both from 2006 to 2008 and from 2009 to 2012 (Table 4). Declines between 2006 and 2008 were similar for men and women. However, those taking place after 2009 were more pronounced in men. All age groups, except those aged 55-64 years, reduced their transportation physical activity considerably between 2006 and 2008. From 2009 onwards, reductions were less marked, with larger declines in younger than older adults. A similar pattern was observed for schooling; all groups reduced their transportation physical activity considerably between 2006 and 2008, while reductions between 2009 and 2012 were less marked, with larger declines occurring among those with low schooling. The adjusted analyses confirmed all findings from the unadjusted one (Table 5).

**Table 4:** Time trends in transportation physical activity* by sex, age and years of schooling- Brazil 2006-2012

**Variables**	**2006**	**2007**	**2008**	**Annual change (%)**	**p value**	**Annual change (%)**	**p value**
Sex							
Male	13.5	12.7	10.3	-12.3	< 0.001	-7.7	< 0.001
Female	8.7	9.1	6.4	-13.7	< 0.001	-3.7	0.017
Age (years)							
18-24	11.4	11.3	9.1	-10.4	0.026	-8.7	0.004
25-34	12.4	12.3	8.4	-16.8	< 0.001	-4.2	0.070
35-44	12.9	13.1	10.2	-10.4	0.002	-6.9	0.001
45-54	12.3	11.7	8.9	-14.7	< 0.001	-6.1	0.008
55-64	7.1	7.5	6.9	-1.7	0.810	-2.5	0.448
65+	3.3	2.3	2.0	-22.6	0.049	+6.1	0.353
Years of schooling							
0-4	13.1	11.2	8.8	-17.8	< 0.001	-7.3	0.029
5-8	13.8	13.5	10.9	-11.1	0.001	-7.2	0.002
9-11	10.4	10.8	8.2	-11.8	< 0.001	-5.3	0.001
12+	6.4	6.8	4.6	-14.4	0.002	-2.0	0.425

*Walking or cycling at least 30 minutes per day to/from work.

**Table 5:** Adjusted analysis on time trends in transportation physical activity* by sex, age and years of schooling- Brazil 2006-2012

**Variables**	**2006**	**2007**	**2008**	**2009**	**2010**	**2011**	**2012**
Sex							
Male	1.00	1.00	1.00	1.00	1.00	1.00	1.00
Female	0.67 (0.61-0.74)	0.75 (0.67-0.83)	0.64 (0.57-0.73)	0.77 (0.70-0.84)	0.78 (0.71-0.86)	0.84 (0.76-0.93)	0.86 (0.77-0.96)
Age (years)							
18-24	1.00	1.00	1.00	1.00	1.00	1.00	1.00
25-34	1.02 (0.87-1.20)	1.02 (0.88-1.20)	0.89 (0.73-1.08)	1.13 (0.94-1.33)	1.17 (1.00-1.37)	1.20 (1.02-1.41)	1.35 (1.12-1.63)
35-44	0.98 (0.84-1.15)	0.99 (0.85-1.16)	0.97 (0.80-1.19)	1.03 (0.87-1.21)	1.19 (1.02-1.39)	1.11 (0.94-1.31)	1.18 (0.98-1.42)
45-54	0.85 (0.72-1.02)	0.84 (0.71-1.00)	0.79 (0.64-0.98)	0.95 (0.80-1.14)	1.08 (0.92-1.27)	0.99 (0.84-1.18)	1.16 (0.96-1.42)
55-64	0.46 (0.37-0.58)	0.49 (0.39-0.61)	0.55 (0.42-0.72)	0.57 (0.46-0.70)	0.67 (0.54-0.82)	0.64 (0.52-0.78)	0.79 (0.64-0.99)
65+	0.20 (0.14-0.27)	0.14 (0.09-0.20)	0.15 (0.10-0.24)	0.16 (0.12-0.23)	0.15 (0.11-0.19)	0.21 (0.15-0.28)	0.27 (0.20-0.37)
Years of schooling							
0-4	1.00	1.00	1.00	1.00	1.00	1.00	1.00
5-8	0.85 (0.73-0.98)	0.92 (0.77-1.09)	0.97 (0.80-1.16)	1.02 (0.85-1.22)	1.13 (0.94-1.35)	1.22 (1.02-1.46)	1.06 (0.87-1.30)
9-11	0.55 (0.48-0.64)	0.64 (0.54-0.77)	0.65 (0.54-0.78)	0.72 (0.60-0.87)	0.85 (0.71-1.02)	0.90 (0.76-1.07)	0.84 (0.68-1.03)
12+	0.36 (0.30-0.44)	0.43 (0.35-0.52)	0.38 (0.30-0.47)	0.55 (0.45-0.68)	0.72 (0.59-0.87)	0.80 (0.66-0.96)	0.71 (0.57-0.88)

*Walking or cycling at least 30 minutes per day to/from work.

Table 6 presents time trends of television time according to sex, age and schooling. In the first period (2006-2009), declines were similar in across subgroups defined by these characteristics. From 2010 to 2012, however, a decline was observed among men, but not among women. In terms of age and schooling, none of the subgroups showed statistically significantly changes (decline or increase) in the second period analysed (2010-2012).

**Table 6:** Time trends in television viewing time* according sex, age and years of schooling- Brazil 2006-2012

**Variables**	**2006**	**2007**	**2008**	**2009**	**Annual change (%)**	**p value**	**Annual change (%)**	**p value**
Sex								
Male	27.6	26.0	24.6	23.7	-5.0	< 0.001	-4.3	0.013
Female	31.3	28.9	27.2	26.7	-5.3	< 0.001	0.0	0.453
Age (years)								
18-24	33.5	34.3	28.1	28.0	-7.1	< 0.001	-1.5	0.586
25-34	28.5	27.1	26.4	25.4	-3.5	0.011	-1.8	0.455
35-44	27.3	24.4	22.6	23.0	-5.8	< 0.001	+1.0	0.693
45-54	27.2	24.8	24.3	23.2	-4.9	0.003	-2.6	0.338
55-64	28.9	26.1	27.1	24.7	-4.2	0.021	-3.2	0.266
65+	34.7	28.5	29.8	28.7	-5.2	0.001	-2.0	0.427
Years of schooling								
0-4	30.3	25.1	25.4	25.8	-4.9	0.004	+1.7	0.582
5-8	31.9	30.2	30.1	29.1	-2.8	0.048	-1.3	0.605
9-11	33.0	31.3	28.7	27.7	-6.0	< 0.001	-1.5	0.348
12+	21.1	20.1	17.8	16.8	-7.7	< 0.001	-3.9	0.091

*More than 3 hours per day watching television.

Table 7 displays adjusted results for the correlates of television viewing time. Overall, findings are highly consistent with those presented in the unadjusted analyses: (a) females were more likely to watch TV for 3+ hours per day between 2006 and 2009, and this differences was no longer observed since 2010; (b) lower levels of TV viewing were observed among those aged 25-34 and 35-44 years; (c) high schooling participants (12+ years) were less likely than the remaining groups to watch 3+ hours of TV per day.

**Table 7:** Adjusted analysis on time trends in television viewing time* by sex, age and years of schooling- Brazil 2006-2012

**Variables**	**2006**	**2007**	**2008**	**2009**	**2010**	**2011**	**2012**
Sex							
Male	1.00	1.00	1.00	1.00	1.00	1.00	1.00
Female	1.14 (1.08-1.21)	1.13 (1.06-1.19)	1.11 (1.04-1.18)	1.14 (1.07-1.21)	0.89 (0.84-0.95)	0.93 (0.88-0.98)	1.00 (0.93-1.06)
Age (years)							
18-24	1.00	1.00	1.00	1.00	1.00	1.00	1.00
25-34	0.84 (0.77-0.91)	0.78 (0.71-0.85)	0.92 (0.84-1.02)	0.88 (0.80-0.97)	0.94 (0.85-1.03)	0.92 (0.84-1.01)	0.93 (0.83-1.03)
35-44	0.78 (0.72-0.85)	0.68 (0.62-0.74)	0.76 (0.69-0.83)	0.76 (0.70-0.84)	0.77 (0.70-0.86)	0.78 (0.71-0.86)	0.79 (0.71-0.89)
45-54	0.76 (0.69-0.83)	0.68 (0.62-0.74)	0.79 (0.71-0.88)	0.73 (0.66-0.82)	0.83 (0.74-0.92)	0.82 (0.74-0.90)	0.78 (0.70-0.87)
55-64	0.78 (0.70-0.86)	0.69 (0.62-0.72)	0.86 (0.77-0.96)	0.75 (0.67-0.84)	1.00 (0.89-1.11)	0.86 (0.78-0.96)	0.92 (0.82-1.03)
65+	0.91 (0.82-1.00)	0.74 (0.67-0.83)	0.90 (0.81-1.01)	0.82 (0.73-0.82)	1.02 (0.92-1.14)	0.98 (0.88-1.09)	0.95 (0.84-1.06)
Years of schooling							
0-4	1.00	1.00	1.00	1.00	1.00	1.00	1.00
5-8	1.05 (0.96-1.14)	1.15 (0.96-1.14)	1.19 (1.08-1.31)	1.10 (0.98-1.23)	1.21 (1.08-1.34)	1.24 (1.12-1.38)	1.11 (0.99-1.25)
9-11	1.07 (0.99-1.16)	1.16 (0.99-1.16)	1.13 (1.03-1.23)	1.02 (0.91-1.14)	1.21 (1.10-1.34)	1.22 (1.11-1.34)	1.09 (0.99-1.21)
12+	0.69 (0.62-0.76)	0.75 (0.62-0.76)	0.70 (0.63-0.78)	0.62 (0.55-0.70)	0.92 (0.83-1.03)	0.89 (0.80-0.99)	0.79 (0.71-0.89)

*More than 3 hours per day watching television.

## Discussion

Few low and middle income countries have surveillance systems that monitor trends in physical inactivity. A 2012 publication highlighted the need for concerted efforts to monitor physical activity in a consistent manner globally, as well as indicated that differential declines in physical activity and increases in sedentary time across the globe represent a major threat to global health [15]. Our study examines changes in leisure time physical activity, transportation physical activity and television viewing time over a recent six-year period in Brazil. The results show both favourable and unfavourable trends. On the one hand, leisure time physical activity increased, and television viewing time decreased, primarily among the youngest and most educated. On the other, transportation physical activity is decreasing in all population subgroups.

Most of the available studies on time trends in leisure time physical activity have come from high income countries. Stamatakis et al [16], using data from Health Survey for England, showed an overall increase in physical activity over time. Regular participation in sports and exercise (defined as more than once a week) increased from 40.8% in 1997/98 to 41.2% in 2006 among men, and 31.2% to 33.9% among women. Increases were larger in older (45+ years) than younger adults. Other studies carried out in Canada [17,18] and the United States [19] also have shown modest increases in leisure time physical activity.

One of the likely reasons for the increase in leisure-time physical activity that we observed over time in Brazil is a strong commitment from the Ministry of Health towards reducing the burden of non-communicable diseases. In 2006, a National Health Promotion Policy was launched [20], and in 2012, the Brazilian Strategic Action Plan to Combat Chronic Non-communicable Diseases by 2022 was released [21,22]. Both documents highlighted physical activity as a crucial area for improvement. In order to achieve such a goal, the Brazilian Ministry of Health is funding over 2,000 municipalities countrywide to develop community physical activity interventions, particularly by offering physical activity classes at no cost to the population and facilitating access to physical activity facilities. Such interventions also led to environmental improvements by reengineering public spaces and providing them with equipment for physical activity practice [23]. In addition, market research has estimated that the number of gym clubs in Brazil has grown more than three-fold in the last decade [24]. All these activities combined also probably increased the population awareness on the importance of physical activity for health.

With regard to the decline in transportation physical activity, this is likely a consequence of the economic growth of the country. In recent years, Brazil has achieved marked improvements in the proportion of the population that is educated, resulting in a better socioeconomic profile. For example, while the Brazilian population increased by 12% between 2000 and 2010 [25], the number of vehicles increased by around 140% between 2001 and 2012 [26].

Sedentary behaviour has emerged as a potential cardiovascular risk factor, being associated with all-cause and cardiovascular mortality [27,28]. Television viewing time is widely used as an indicator of sedentary behaviour. In the period evaluated in our analyses, the proportion of adults reporting more than three hours watching television has consistently decreased. In spite of this change, it is important to highlight that television viewing time only represents a small fraction of total time spent in sedentary behaviour [29]. Although television viewing time decreased in the 6-years period, there is a possibility that people are replacing television time with other forms of sedentary behaviour. In fact, in a previous study we showed that TV viewing is still the predominant source of sedentary behaviour among the poor in Brazil, but among the better-off, it has a much lower contribution to total sedentary time [29]. Chau et al. [30] using data from Australian Time Use Surveys, showed an increase in leisure-time computer use and changes in patterns of time engaged in different types of sedentary behaviour between 1992 and 2006.

Some limitations should be taking into account when interpreting our findings. According to the countrywide census carried out in 2010, there were 45,475,045 inhabitants in the state capitals, representing 23.8% of the total Brazilian population. Thus, we need to be cautious when extrapolating the observed time trends here to the rest of the country. Another limitation is that sampling was based on telephone land line coverage in the state capitals. We tried to address this limitation by using statistical strategies to weight analyses by socio-demographic composition. A third limitation is that questions on transportation physical activity and television viewing time were not identical over the years. However, we attempted to create indicators that were comparable for these two variables. Finally, our physical activity indicators did not take the intensity of activities into account, i.e. all activities had the same weight in the creation of physical activity scores in minutes per week.

We opted not to evaluate time trends in household and occupational physical activity because questions changed over time in VIGITEL and the instrument does not quantify minutes per week spent on activities. Additionally, physical activity in these domains is strongly related to socioeconomic status and thus might not reflect an individual behavioural choice. One might argue that transportation physical activity is also related to socioeconomic status. However, this is likely more amenable to individual choice if policies that promote urban restructuration and a safe environment (e.g., bicycle lanes) are available, giving options for commuting other than using cars and motorcycles.

In summary, national data from a large middle income country showed increases in leisure time physical activity and decreases in television viewing time over a recent 6-year period. However, transportation physical activity declined. Marked changes in physical activity indicators according level of schooling are widening inequalities in physical activity over time practice. These data are useful for informing public health policies. It is important to continue to monitor physical activity levels in the country, as well as to develop policies that promote active transport.
